# High natural UVB radiation regulates grain pigmentation in black rice: insights from a genome-wide association study

**DOI:** 10.3389/fpls.2026.1943027

**Published:** 2026-10-30

**Authors:** Truong Duc Nguyen, Manisha Thapa, Erwin Tandayu, Lei Liu, Szabolcs Lehoczki-Krsjak, Tobias Kretzschmar

**Affiliations:** 1 Faculty of Science and Engineering, Southern Cross University, Lismore, NSW, Australia; 2 Agriculture Victoria, Melbourne, VIC, Australia

**Keywords:** anthocyanins, black rice, functional food, genome-wide association study, grain pigmentation, ultraviolet, UV-B response

## Abstract

**Introduction:**

Black rice gains recognition in functional food and nutraceutical for its secondary metabolite profiles, which exhibit antioxidant, anti-diabetic, anti-hyperlipidemic, and anti-cancer properties. Its unique purple-black color trait results from anthocyanin accumulation in the pericarp, a trait that varies across pigmented genotypes. While the genetic basis of grain pigmentation in black rice has been partially elucidated, the influence of environmental factors, particularly ultraviolet (UV) radiation, remains poorly understood. UVB radiation is thought to play a role in regulating anthocyanin regulation in various crops, suggesting a potential advantage for cultivating black rice in high UV regions. This research aimed to identify quantitative trait loci (QTL) linking grain pigmentations in response to UV exposure.

**Methods:**

A genome-wide association study (GWAS) was performed on a diversity panel of 191 black rice accessions primarily sourced from the Asia-Pacific region.

**Results:**

Utilizing 31,501 single-nucleotide polymorphisms (SNPs), GWAS revealed distinct associations for anthocyanin content under UV conditions versus under non-UV conditions. Notably, three putative candidate genes associated with anthocyanin biosynthesis under UV exposure were identified: *OsBBX14*, known to activate *OsC1* or *OsB2* in anthocyanin biosynthesis pathway; *OsMYB44*, a transcription factor that promotes UV-B tolerance; and *OsbZIP71*, which improves abiotic stress tolerance by reducing reactive oxygen species (ROS) accumulation.

**Discussion:**

These findings provide critical insights into the genetic associations regulating grain pigmentation under contrasting UV regimes and pinpoint potential candidate genes for further validation and molecular marker development. Ultimately, this research supports the development of black rice varieties with enhanced nutritional value and improved resilience to high-UV growing conditions.

## 1 Introduction

Black rice, often referred as purple rice or imperial rice, holds deep cultural and historical significance throughout Asia. It has been cultivated for centuries in several Asian countries, including China, Indonesia, Philippines and Thailand (Kushwaha, 2016). Its distinctive dark hue color makes it a popular ingredient in desserts, porridge and black rice cakes. In traditional medicine, black rice has been incorporated as remedy for various health conditions, digestive disorders, diabetes and high blood pressures among others (Mbanjo et al., 2020; Das et al., 2023; Rahim et al., 2022). Several recent studies support the health claims of black rice, attributing them to its anti-oxidant, anti-diabetic, anti-microbial and anti-cancer properties (Deng et al., 2013).

Since no record of the black grain color trait are found in the ancestral wild species *Oryza rufipogon*, it is suspected that the black color on grains emerged during or after the rice domestication (Oikawa et al., 2015). One potential explanation lies in the geographical location where the rice cultivation took place, which influenced their metabolite profile. Rice cultivated in areas with higher ambient UVB radiation or colder climates experience oxidative stress due to reactive oxygen species (ROS) production (Hidema and Kumagai, 2006; Bonnecarrère et al., 2011). In order to mitigate these abiotic stresses, rice plants synthesize phenylpropanoid compounds with antioxidant properties, particularly anthocyanin, which accumulates in the vacuoles of plant cell (Passeri et al., 2016). The accumulation of anthocyanin results in the characteristic black or purple pigmentation observed in vegetative parts and grains of rice plants (Das et al., 2023).

The predominant anthocyanin in rice grains include cyanidin-3-*O*-glucoside, peonidin-3-*O*-glucoside, cyanidin-3-rutinoside and cyanidin-3-*O*-galactoside, with cyanidin-3-*O*-glucoside (C3G) being the most abundant, accounting for 64-90% of total anthocyanin content (Deng et al., 2013; Mackon et al., 2021a). Similar to other flavonoids, the key enzymes involved in anthocyanin biosynthesis in rice have been well characterized (Jez et al., 2000; Kim et al., 2008; Shimizu et al., 2012). However, unlike the model crop Arabidopsis, where each enzyme is typically encoded by a single gene, several enzymes in the anthocyanin pathways in rice are encoded by multiple structural genes (Mbanjo et al., 2020). Through a genome-wide identification and expression analysis, 85 key structural genes associated with the flavonoid biosynthesis in rice were identified and classified into 13 gene families encoding enzymes in the pathway (Wang et al., 2022). During anthocyanin biosynthesis, proteins from different families may form macromolecular complexes, highlighting the complexity of anthocyanin pathway regulation in rice. The activation of these structural genes is regulated by the MYB-bHLH-WD40 (MBW) complex, which consists of different clusters of regulatory proteins that control tissue-specific anthocyanin pathway (Mackon et al., 2021a). Two key transcription genes, *Kala 3* (Os03g0410000) and *OsC1* (Os06g0205100), encode MYB, the main component of this complex (Maeda et al., 2014; Gao et al., 2011). The gene *Kala 4* or *OsB2*, located in locus Os04g0557500, encode the transcription factor basic-helix-loop-helix (bHLH) (Sakamoto et al., 2001; Maeda et al., 2014) while the WD40 protein, which interacts with bHLH and involves in DNA binding, is encoded by the gene *OsTTG1* (Os02g0682500) (Yang et al., 2021). *OsC1* and *OsB2* can be activated by *OsBBX14* (Os05g0204600) and *OsHY5* (Os06g0601500), either collaboratively or independently (Kim et al., 2018).

The complexity of anthocyanin biosynthesis in rice is further compounded by its interaction with environmental signals such as UV-B and cold climates (Lafountain and Yuan, 2021). Metabolic and transcriptomic profiling revealed that the regulation of phenylpropanoids, tyrosine and tryptophan biosynthesis are essential for UV-B response and tolerance in rice (Zhang et al., 2024). Recent work has identified some candidate genes involved in the UV-induced flavonoids biosynthesis, including *OsCHS8*, *OsUVR8b*, *OsMYB44*, and *OsbZIP18* (Liu et al., 2024; Zhang et al., 2024; Park et al., 2020). Among them, *OsCHS8* (Os07g0214900) contributes to UVB tolerance by facilitating the production of secondary metabolite sakuranetin in response to UV (Park et al., 2020). *OsUVR8b* (Os04g0435700) plays an important role in both flavonoid biosynthesis and UV-B tolerance (Chen et al., 2024; Ashokkumar et al., 2024). *OsMYB44* (Os09g0106700) acts as a positive regulator for UV-B tolerance in rice through its involvement in tryptamine biosynthesis (Zhang et al., 2024) while a HY5 ortholog, *OsbZIP18* (Os02g0203000), positively regulates the phenylpropanoids and flavonoid biosynthesis (Liu et al., 2024).

Efforts to unravel the genetic basis of anthocyanin biosynthesis and pigmentation in rice grains have been utilized association mapping or genome-wide association study (GWAS) approaches (Wang et al., 2023; Mbanjo et al., 2023; Kiran et al., 2025). Grain pigmentation is commonly assessed by using CIELAB color parameters, where L represents lightness, a indicates the green-to-magenta axis, and b represents the blue-to-yellow axis. CIELAB values have been shown to correlate moderately with anthocyanin content (Liang et al., 2011; Cesa et al., 2017; Mbanjo et al., 2023). A notable GWAS by Mbanjo et al. (2023), which investigated phenolic compounds and grain pigmentation across 376 diverse rice accessions, identified the gene *Rc/bHLH17* - known to regulate the biosynthesis of proanthocyanidin (Sweeney et al., 2006) - as being associated with CIELAB a and b parameters. Similarly, two other GWAS studies investigating grain pigmentation also pinpointed *Rc* as a key candidate gene associated with CIELAB values (Wang et al., 2023; Kiran et al., 2025). However, *Rc* is primarily responsible for red pigmentation in rice grains (Sweeney et al., 2006), suggesting that CIELAB values may reflect the presence of other compounds beyond anthocyanin and may not serve as reliable indicators of anthocyanin levels. To address this limitation, Mbanjo et al. (2023) also conducted a GWAS analysis on quantification of anthocyanins, namely cyanidin glycoside and peonidin glycoside, using High-Performance Liquid Chromatography (HPLC). However, this study did not identify any significant candidate genes. To date, no GWAS studies have examined the genetic mechanisms regulating anthocyanin biosynthesis in pigmented rice in response to UV-B stress.

In this study, a black rice (*Oryza sativa* L. ssp. *japonica*) diversity panel, consisting of 191 varieties, was utilized in a field study with selective UV-blocking. Anthocyanin content was precisely quantified using HPLC and the trait-loci correlation was analyzed with GWAS. The most advanced multi-locus and single-locus GWAS models were employed through GAPIT platform. The analysis identified several significant marker-trait associations near known and potential regulatory genes involved in anthocyanin biosynthesis, seed pigmentation and UV-B response, thereby expanding the current understanding of how environmental factors influence grain pigmentation in rice. These putative candidate genes serve as key targets for further validation and marker development, contributing to breeding efforts aimed at enhancing nutritional content and UV-B resilience in rice.

## 2 Materials and methods

### 2.1 Plant materials and experimental design

The diversity panel, consisting of 191 inbred *japonica* black rice landrace accessions primarily sourced from Asia-Pacific region, was obtained from the International Rice Research Institute and selected for this study (**Supplementary Table S1**). Independent assessment of seed purity was not performed for each accession. However, heterozygosity rate estimated from the Genotyping-By-Sequencing (GBS) data provides an indication of genetic uniformity within the 163 accessions for which GBS data were generated (**Supplementary Table S12**).

The field experiment was conducted at the Southern Cross University rice nursery located at Lismore, New South Wales, Australia (GPS: 28°49’37.2”S 153°17’58.4”E) from December 2021 to June 2022. A randomized resolvable incomplete block design (augmented design) was generated using the Breeding Management System (Integrated Breeding Platform), with non-replicated accessions and replicated checks. For each plot, three biological replicates were collected as subsamples from the same plot.

Two UV regimes were established in this trial (**Supplementary Figure S6**). For ambient UV condition, plants were grown under the full exposure to natural UV radiation. For non-UV conditions, an UV blocking structure was constructed at the onset of flowering, covered with Bastion clear polycarbonate roofing sheets that block UV radiation while permitting full light transmission. To ensure rainwater penetration under UV filtering structure, 5mm diameter holes were drilled through the polycarbonate sheets at 10 cm intervals. Significant rainfall throughout the growing season, combined with two major floods in the area, allowed water availability to remain consistent across both conditions (**Supplementary Table S11**). UV-B measurements by SOLARMETER Model 6.2 (Solar Light Company, Inc., USA) showed a clear difference between treatments. Under the UV blocking structure, UV-B levels ranged from 0 to 35 µW/cm², whereas in the UV-exposed conditions, it ranged from 184 to 200 µW/cm² measured in the intersections of a 0.2m x 1m cell grid covering the whole experimental area (**Supplementary Table S13**). Flowering dates were recorded for each plot. Following the Australian rice growing guidelines for harvesting time (Ward, 2021), three biological replicates were harvested as subsamples from each plot at 53 days after flowering. The seeds were then dehulled for grain morphology assessment with CSIRO GrainScan v3 software (Whan et al., 2014).

### 2.2 Anthocyanin quantification

Seed samples were dried in a cool room at 15˚C and 15% relative humidity until 12-13% of moisture content was reached. One gram of seeds from each biological replicate of each genotype was dehusked with a hand dehusker and ground into a fine powder using a Retsch Mixer MM301 ball mill. Anthocyanin extraction and quantification followed the method described by Thapa et al. (2024). For extraction, 50 mg seed powder from each biological replicate was placed in a 2 mL Eppendorf tube and mixed with 1 mL of an extraction solvent composed of methanol acidified with 1M HCl (85:15, v:v). The mixture was sonicated for 30 minutes in a Soniclean Ultrasonic cleaner and then centrifuged at 18000 rcf for 10 minutes. The resulting supernatant was transferred into Agilent HPLC vials for analysis. The anthocyanin quantification was performed using an Agilent 1260 Infinity II-High Performance Liquid Chromatography instrument, equipped with an Agilent C18 reverse phase column (50mm x 2.1 mm, 1.8 μm). The mobile phase consisted of 100% MilliQ water with 0.05% Trifluoroacetic acid as solvent A and 100% acetonitrile with 0.05% Trifluoroacetic acid as solvent B. UV absorbance was detected at 520nm for cyanidin-3-*O*-glucoside (C3G) and peonidin-3-*O*-glucoside (P3G). Representative chromatograms of C3G and P3G were provided in **Supplementary Figure S1**. Quantification was conducted with a calibration curve created using standard C3G obtained from Phytolab. P3G concentration was estimated using the same C3G calibration curve. Total anthocyanin content was calculated as the sum of C3G and P3G concentrations.

### 2.3 Genotyping-By-Sequencing and quality control

Leaf tissues from 3 to 5 plants per accession were collected at 60 days after sowing for DNA extraction using the standard Qiagen Mini-Prep DNA Extraction kits. The DNA samples were sent for Genotyping-By-Sequencing with SNP calling performed by The Elshire Group Ltd (Palmerston North, NZ) (Elshire et al., 2011; Tange, 2011). Library preparation was carried out by The Elshire Group Ltd, and paired-end sequencing (2x of 150 bp reads) was conducted on a single lane of a HiSeq sequencing platform (Illumina, USA). Sequence outputs were demultiplexed using Kevin Murray’s axe-demux (Murray and Borevitz, 2018). Both reads from the paired-end data were aligned against the Osativa_323_v7.0 genome reference (Ouyang et al., 2007), and SNP calling was performed using the GBSV2 pipeline (Glaubitz et al., 2014) implemented in TASSEL 5.0 (Bradbury et al., 2007). Quality control on the SNP data was carried out in TASSEL 5.0 to retain only SNPs with minor allele frequency >0.05, maximum heterozygosity proportion <0.2, proportion of missing genotypes < 0.2. Samples were filtered to include only those with maximum of 0.2 missing genotypes. Imputation was performed using the LD KNNi method with parameters set to high LD sites at 30, number of nearest neighbors at 30 and maximum distance between site to find LD at 10,000,000.

Population structure of the panel was analyzed in relation to a collection of modern white rice cultivars (**Supplementary Table S2**). An Unweighted Neighbor-joining phylogenetic tree was constructed using DARwin (Perrier and Jacquemoud-Collet, 2006), based on the Identity by State distance calculated from SNP data on TASSEL 5.0. Principal Component Analysis (PCA) was performed in TASSEL 5.0 and visualized using the “ggplot” package in R (Wickham, 2016).

### 2.4 Statistical analysis and variance component analysis

Single-site statistical analysis was performed using the Breeding View software (VSN International, Hemel Hempstead, UK). A mixed-model approach was used to account for the randomized resolvable incomplete-block design. Variance components were estimated using Restricted Maximum Likelihood (REML), with genotype fitted as a random term. Best Linear Unbiased Predictions (BLUPs) were subsequently obtained from the mixed model and used as the genotype-level phenotypic values for the GWAS analyses.

### 2.5 Association mapping

Association mapping was conducted using Genomic association and prediction integrated tool package (GAPIT) version 3 in R (Wang and Zhang, 2021). The analysis employed three advanced multi-locus test methods: multiple locus mixed linear model (MLMM), Fixed and random model Circulating Probability Unification (FarmCPU), Bayesian-information and Linkage-disequilibrium Iteratively Nested Keyway (BLINK), along with the single-locus test method, Settlement of MLM Under Progressively Exclusive Relationship (SUPER). Quantile-quantile plots generated by GAPIT were used to identify the best model based on its ability to control spurious associations caused by population structure and genetic relatedness. Bonferroni multiple test correction was applied, setting the significance threshold at 5%. The results of the GWAS were visualized in Manhattan plots using “CMplot” package (Yin et al., 2021) in R. Candidate gene analysis was performed 400 kb upstream and downstream of significant SNPs, utilizing the RAP-DB annotation system.

## 3 Results

### 3.1 Genotypic characterization

The black rice diversity panel comprised 191 accessions from 11 countries (**Supplementary Table S1**), with the largest representation from Indonesia (60 genotypes), Philippines (36 genotypes), Lao PDR (33 genotypes) and Malaysia (28 genotypes). The visual assessment of grain colors revealed that 56.5% of the panel exhibited purple grains, 41.9% were variable purple and 1.6% were brown.

SNP calling identified a total of 250,065 SNPs using a standard Genotyping-by-Sequencing (GBS) platform. The final set of SNPs was well distributed across the genome (**Supplementary Figure S2**), with the percentage of SNPs per chromosome ranging from 4.39% on chromosome 9 to 14.01% on chromosome 1 (**Supplementary Table S7**). After filtering to retain SNPs with a minor allele frequency threshold of >0.05, a maximum heterozygosity of < 0.2, and a missing genotype rate of < 0.2, 31,501 high-confidence SNPs remained as input for GWAS analysis. Out of 191 genotypes planted, 163 lines produced sufficient seeds under at least one of UV regimes and were selected for pigmentation trait evaluation. After filtering samples to retain those with > 0.8 proportion of genotypes present, three lines were removed, leaving 160 lines for the final analysis.

The Unweighted Neighbor-joining tree, constructed by DARwin (Perrier and Jacquemoud-Collet, 2006), exhibited clear genetic separation between the majority of black rice panel and the white rice cultivars (**Supplementary Table S2**), although some degree of overlap was observed (**Figure 1A**). Principal Component Analysis (PCA) of the genotypic data showed that the first principal component explained 25.86% of total genetic variation, effectively separating two distinct clusters of black rice lines (**Figure 1B**). The second component accounted for only 5.64%, primarily capturing the genetic differentiation between black rice panel and white rice cultivars. A small number of white rice cultivars were close to the black rice group. This clustering reflects some degree of genetic relatedness and ancestry. While the clustering patterns in both phylogenetic tree and PCA suggested the presence of some substructure within the black rice panel, possibly reflecting differences in geographic origin, the overall distribution of genetic variation appeared continuous without sharply defined subgroup boundaries. This suggested the black rice panel captured a broad representation of genetic variation, making it suitable for association mapping.

**Figure 1:**
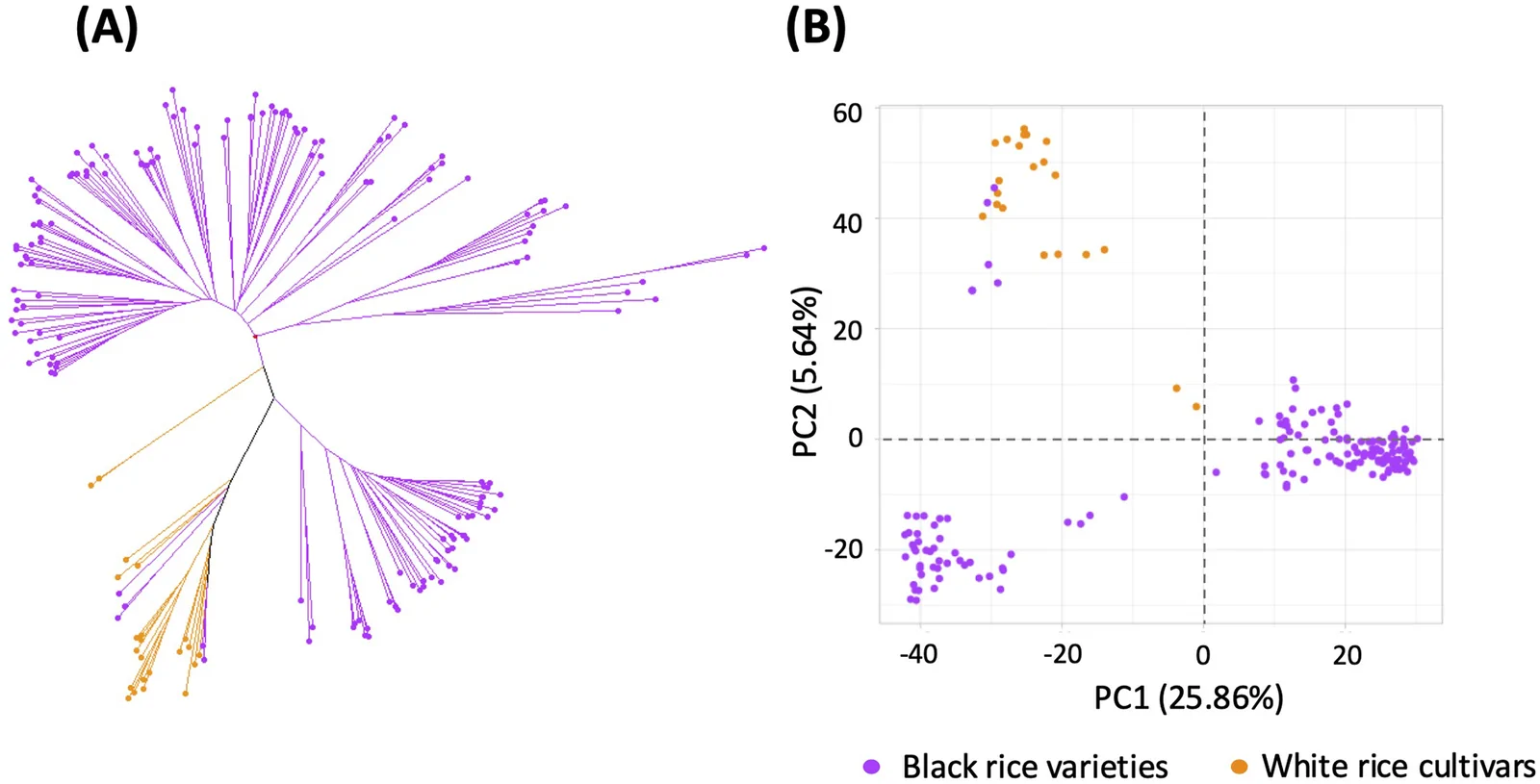
Population structure of black rice varieties from black rice panel compared to modern white rice cultivars: **(A)** Neighbor-joining tree based on genetic distances. Tree was constructed on DARwin 6.0 with branches colored by black rice panel (purple) and modern white rice (orange); **(B)** Principal component analysis conducted in TASSEL 5 and plotted in R.

### 3.2 Descriptive statistics and correlations

The distribution of raw cyanidin-3-*O*-glucoside (C3G), peonidin 3-*O*-glucoside (P3G) and total anthocyanin (**Figures 2A–C**) highlighted the differences in anthocyanin accumulation under both treatments. Under UV treatments, C3G increased in 73 lines, with an average increase of 23.9 mg/100g seed, while 68 lines showed a decrease in C3G with an average reduction of 39.3 mg/100g seed. P3G level rose by an average increase of 3.4 mg/100g seed in 85 lines and declined by 4.6 mg/100g seed in 47 lines. For total anthocyanin, 76 lines accumulated more under UV conditions (average increase of 26.4 mg/100g seed), whereas an average drop of 43.4 mg/100g seed was recorded in 66 lines.

**Figure 2:**
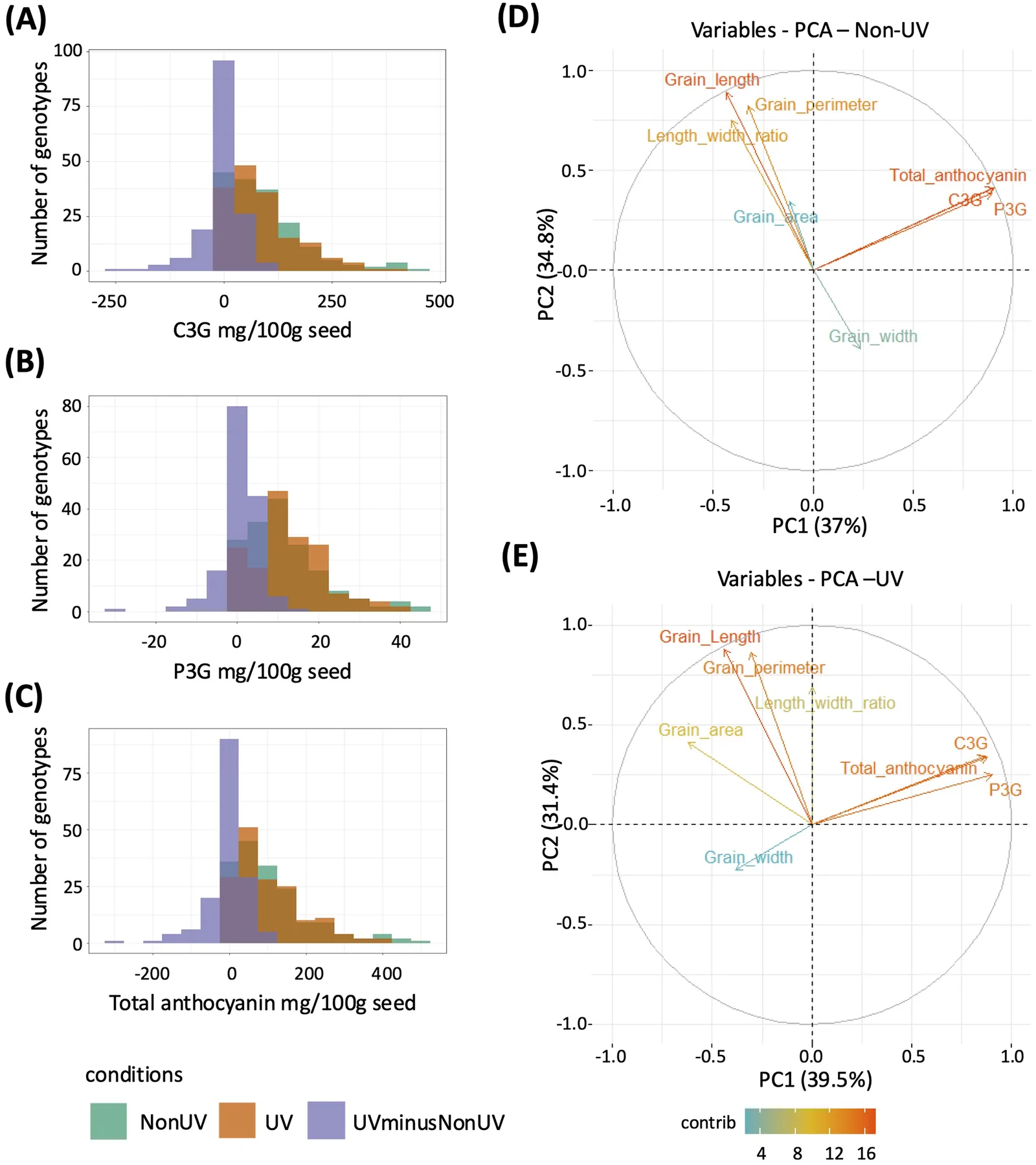
Distribution histogram of raw mean values for grain pigmentation traits under non-UV conditions (green), UV conditions (orange) and difference between the two conditions (purple): **(A)** C3G (mg/100g seed); **(B)** P3G (mg/100g seed); **(C)** total anthocyanin (mg/100g seed). Principal component analysis (PCA) of anthocyanin contents and grain morphology traits with contribution of variables: **(D)** under non-UV conditions; **(E)** under UV conditions.

Under non-UV conditions, C3G showed a higher mean concentration (95.09 mg/100g seed) and a broader range (0.40 - 463.98 mg/100g seed) compared to C3G under UV conditions, which had a mean of 89.90 mg/100g seed and a range of 0.26 - 382.63 mg/100g seed (**Supplementary Table S4**). In contrast, P3G levels were slightly higher under UV conditions (mean = 12.65 mg/100g seed) than under non-UV (mean = 11.89 mg/100g seed), although the concentration range remained wider in the non-UV treatment. Total anthocyanin content followed the similar trend to C3G, with a higher mean under non-UV conditions (106.80 mg/100g seed) than under UV (102.55 mg/100g seed). This pattern aligns with the anthocyanin composition in the grain, where C3G contributed an average of 87.5% of total anthocyanins, compared to 12.5% from P3G (**Supplementary Table S3**). Higher coefficients of variation were observed across all anthocyanin measurements under non-UV conditions, suggesting greater variability in anthocyanin accumulations in the absence of UV exposure. The phenotypic differences observed in C3G, P3G and total anthocyanin across conditions were not statistically significant (**Supplementary Table S4**).

The source of variations of anthocyanin content was further investigated by the variance component analysis with Restricted Maximum Likelihood in Breeding View software. Most of variations in C3G, P3G and total anthocyanin were contributed by genotypes under both UV conditions (**Supplementary Table S4**). The high heritability estimates for these traits, ranged from 0.73 to 0.81, further supported the strong genetic contribution to phenotypic variation.

Principal component analysis (PCA) of anthocyanin content and seed morphology traits revealed considerable phenotypic variations among the genotypes. Under non-UV conditions, the first principal component (PC) explained 37% of variation, mostly contributed by anthocyanin traits, while PC2 accounted for 34.8% of variation, largely driven by seed morphology traits (**Figure 2D**). A similar pattern was observed under UV conditions, where PC1, strongly associated with anthocyanin content, explained 39.5% of total variation, PC2, mainly influenced by seed morphology traits, accounted for 31.4% (**Figure 2E**). Notably, the vectors for anthocyanins and seed morphology traits pointed in opposite directions along the PC1 axis under both conditions, indicating negative relationship between these trait groups.

The connection between anthocyanin content and seed morphology traits were revealed by correlation analysis (**Figure 3**). Strong positive correlations were consistently observed among C3G, P3G and total anthocyanin under both UV conditions, with coefficients ranging from 0.91 to 0.99 (**Supplementary Tables 5**, **6**). The correlation between C3G and total anthocyanin was significantly strong, reflecting the predominant contribution of C3G to total anthocyanin in the grain. Under non-UV conditions, anthocyanins showed negligible correlation with seed morphology traits (r = -0.05 - 0.02), while slightly stronger correlations were observed under UV conditions (r = -0.3 - 0.12). Notably, under UV conditions, P3G exhibited a weak but statistically significant negative correlation with grain length (r = -0.21) and grain area (r = -0.3). This indicates that genotype with higher P3G levels might have slightly shorter and smaller grains under UV exposure. Overall, the correlation analysis indicated the independent relationship between anthocyanin accumulation and seed morphology traits.

**Figure 3:**
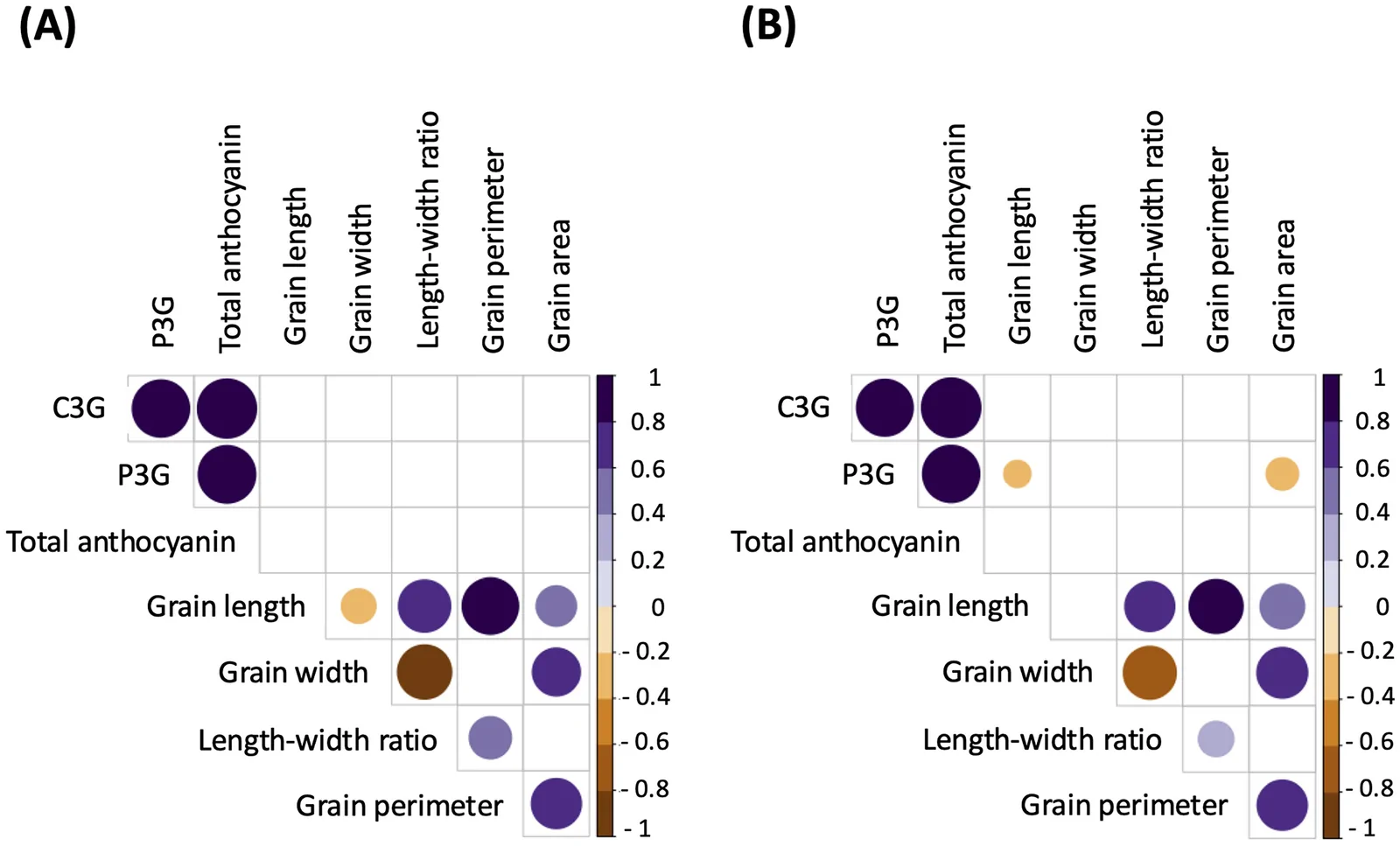
Correlation matrix between anthocyanin contents and grain morphology traits under: **(A)** non-UV condition and **(B)** UV condition. The correlation analysis was computed in R and plotted with corrplot package. Correlation with p value>0.01 (Pearson significance testing) was left blank in the matrix.

### 3.3 GWAS outcomes

Among the models tested, BLINK demonstrated best correction for confounding effects due to population structure and familial relatedness evidenced by very high concordance between observed and expected *p* values for the GWAS of C3G, P3G and total anthocyanin under UV and non-UV conditions (**Supplementary Figure S3**). Similarly, MLMM showed strong correlations for all anthocyanin traits, however, did not found any significant association. FarmCPU returned slightly deflated -log10 p values for C3G and total anthocyanin, indicating possibility of false negatives. In contrast, SUPER displayed inflated p values for all traits, indicating under-correction for confounding effects and high likelihood of spurious associations. Thus, we prioritized characterizing the associations from BLINK being the most robust model, as well as FarmCPU albeit with fewer associations. The Manhattan plots from BLINK (**Figure 4**) and FarmCPU (**Supplementary Figure S4**) displayed high-confidence associated SNPs detected above Bonferroni thresholds (-log10(p) =5.81) for C3G, P3G and total anthocyanin.

**Figure 4:**
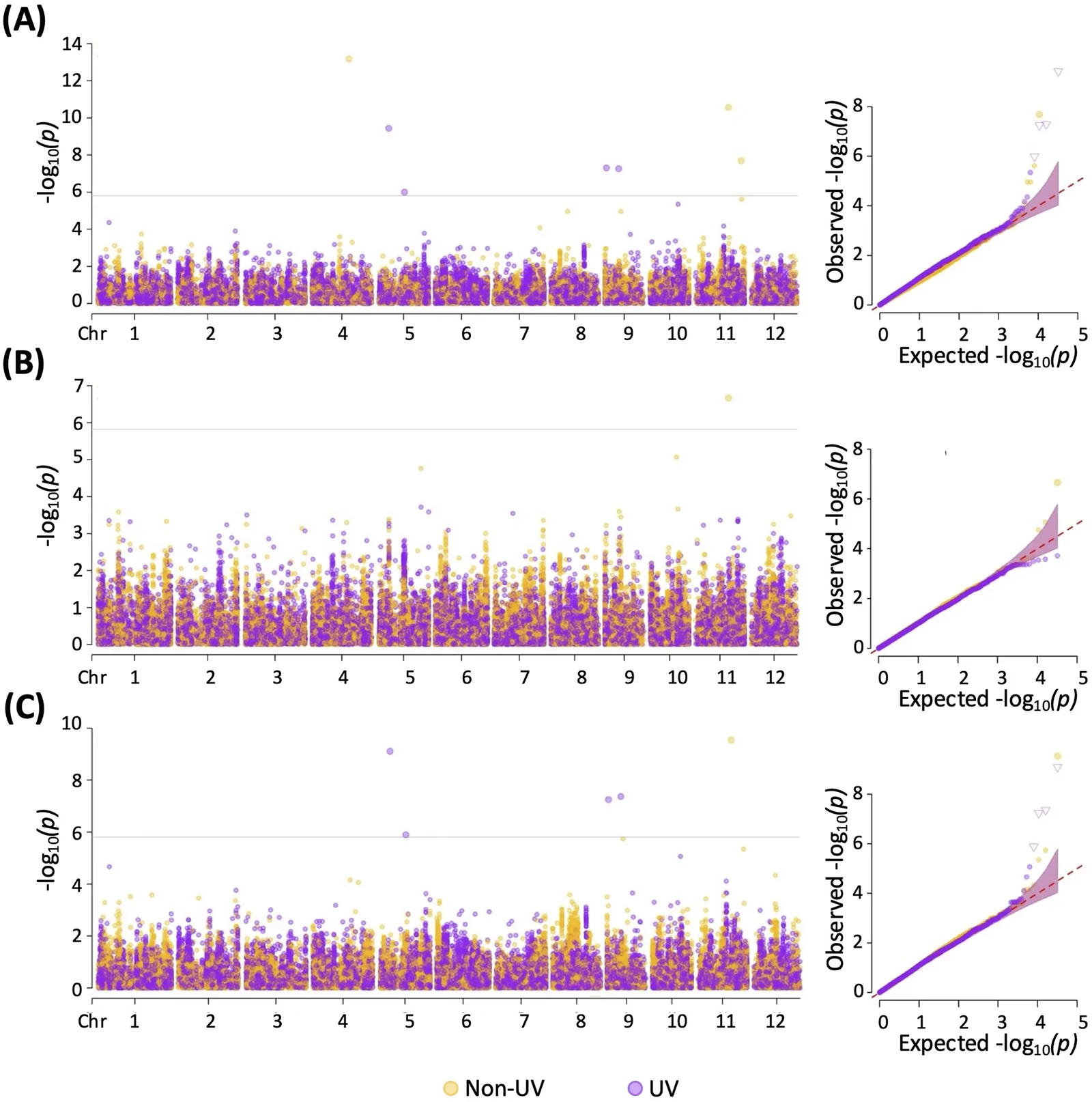
Manhattan and q-q plots for the GWAS of BLINK under non-UV condition (yellow dots) and UV conditions (purple dots): **(A)** C3G; **(B)** P3G; **(C)** total anthocyanin. The gray horizontal line indicates the significance threshold by Bonferroni multiple test correction approach.

Under non-UV conditions, BLINK identified three SNPs associated with C3G, along with one associated SNP each for P3G and total anthocyanin. FarmCPU returned two SNPs associated with C3G and one with total anthocyanin. Under UV conditions, BLINK identified four SNPs common to both C3G and total anthocyanin, reflecting the major contribution of C3G to total anthocyanin content in grains. FarmCPU detected three SNPs linked to C3G, five to P3G and three to total anthocyanin.

Among the most significant associations under non-UV conditions, SNP S11_19582136 showed strong signals across all traits, with a -log_10_*(p)* of 10.6 for C3G, 6.7 for P3G and 9.5 for total anthocyanin (**Supplementary Table S8**). Another SNP, S4_21879467, associated with C3G identified by both BLINK and FarmCPU had a high -log_10_*(p)* of 13.2 in BLINK. Under UV conditions, the strongest association was observed with SNP S5_6240246, detected by BLINK, with -log_10_*(p)* values of 9.4 for C3G and 9.1 for total anthocyanin. Additionally, two SNPs, S9_817645 and S9_8066875, were detected by BLINK as being associated with both C3G and total anthocyanin. Notably, S5_25298114 was identified by FarmCPU for both P3G and total anthocyanin under UV conditions.

### 3.4 Significant SNPs and putative candidate genes

The linkage disequilibrium (LD) decay distance was 424.9 kb when LD coefficient r^2^ reached half of its maximum values (**Supplementary Figure S5**), indicating LD significantly decreased at this distance. Consequently, the candidate gene search was conducted in the range of 400kb upstream and downstream of the associated SNPs with anthocyanin traits. According to annotations from RAPDB and literature, candidate genes were categorized as known or potential UV-inducible anthocyanin regulation genes (**Table 1**; **Supplementary Table S9**).

**Table 1:** Potential candidate genes involved in anthocyanin accumulation under two UV conditions, detected by different GWAS models: BLINK (B), FarmCPU (F), SUPER (S).

Conditions	SNP	Trait	PVE (%)	Model	P-value	Candidate Gene	Distance to SNP (kb)	Candidate Gene
Non-UV	S4_21879467	C3G	53.3	B, F	6.80E-14	Os04g0435500	-215.16	*OsTCHQD1*
Non-UV	S11_19582136	C3G	14.6	B, F	2.76E-11	Os11g0530600	-301.13	*OsCHS24*
		P3G	31.2	B	2.18E-07			
		Total anthocyanin	33.2	B, S	2.88E-10			
UV	S5_6240246	C3G	26.3	B, F	3.69E-10	Os05g0204600	277.03	*OsBBX14*
		Total anthocyanin	25.8	B	7.86E-10			
UV	S5_25298114	P3G	0.8	F	1.36E-08	Os05g0515400	263.67	*OsARF3la*
		Total anthocyanin	4.3	F	5.79E-07			
UV	S9_817645	C3G	6.5	B	5.07E-08	Os09g0106700	-159.27	*OsMYB44*
		Total anthocyanin	6.4	B	5.70E-08			
UV	S9_8066875	C3G	28.5	B	5.58E-08	Os09g0306400	-193.22	*OsBZIP71*
		Total anthocyanin	29.3	B	4.30E-08			

Phenotypic variance explained (PVE) was calculated by GAPIT. When a SNP is detected by more than two models including BLINK, the PVE displayed was from BLINK.

Under non-UV conditions, a significant SNP, S11_19582136, was detected, explaining 14.6% of phenotypic variation (phenotypic variation explained - PVE) in C3G, 31.2% in P3G and 33.2% in total anthocyanin. A known anthocyanin structural gene, *OsCHS24* (also referred to as *OsCHS1*), which encode chalcone synthase for anthocyanin biosynthesis (Park et al., 2020), was located 301.13 kb downstream of S11_19582136. A putative structural gene involved in anthocyanin transport and accumulation, *OsTCHQD1* (Jain et al., 2010) was found 215.16 kb downstream of SNP S4_21879467, which showed a high PVE of 53.3% for C3G. The distribution of phenotypic means for genotypes with different alleles of S11_19582136 was shown in **Figure 5B**, where genotypes carrying the A allele accumulated more total anthocyanin content than those carrying the G allele. The C allele at S4_21879467 was indicative of lower total anthocyanin content than the T allele.

**Figure 5:**
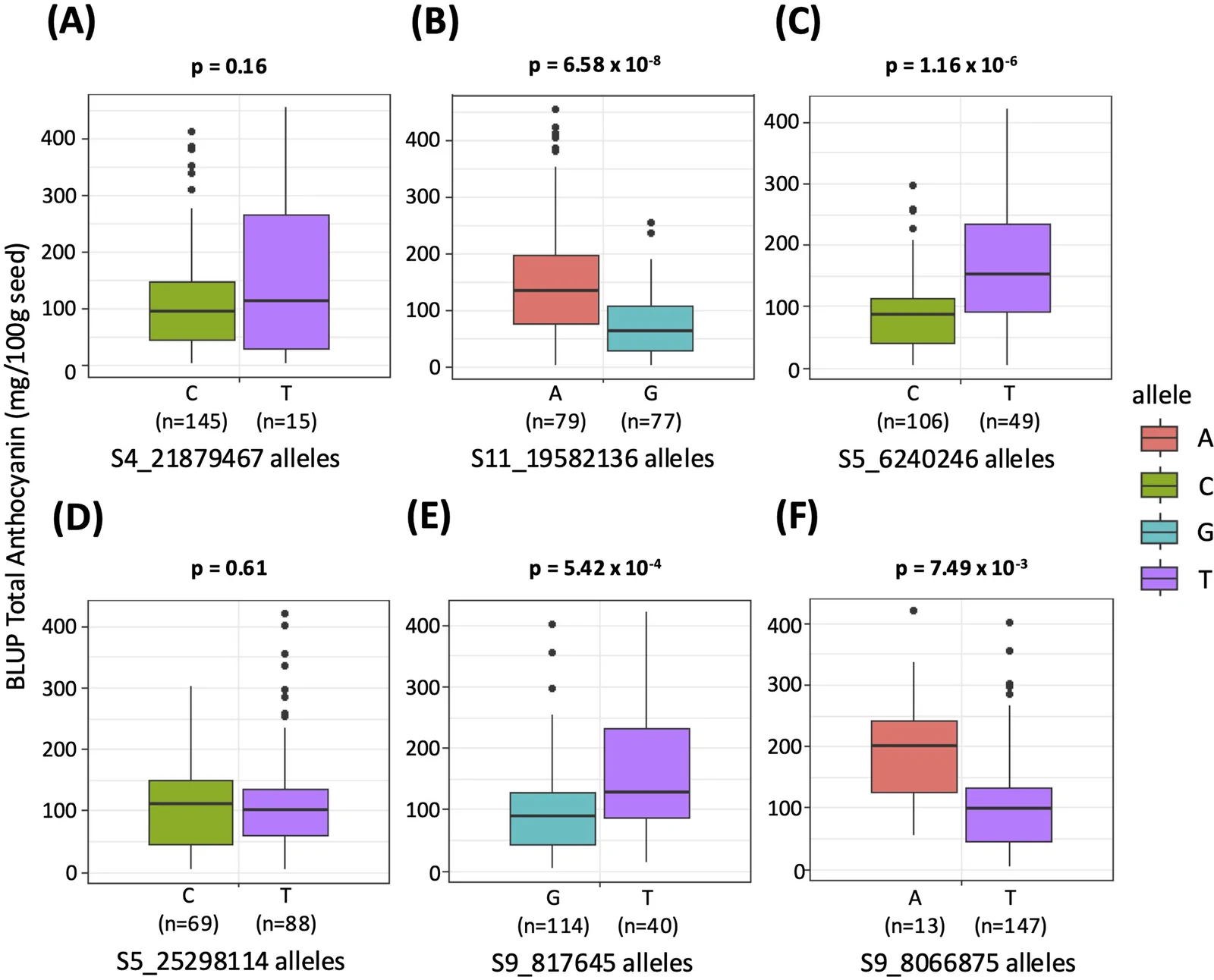
Means comparison of genotypes carrying different alleles at SNPs significantly associated with BLUPs of total anthocyanin content used in GWAS. Representative SNPs include: **(A)** S4_21879467 and **(B)** S11_19582136 non-UV conditions; **(C)** S5_6240246, **(D)** S5_25298114, **(E)** S9_817645 and **(F)** S9_8066875 under UV conditions. P-values were calculated using t-test with the assumption of unequal variances between two genotype groups.

Under UV conditions, *OsBBX14*, a known anthocyanin regulation gene that activates *OsC1* or *OsB2* in anthocyanin biosynthesis (Kim et al., 2018), was found 277.03 kb upstream of SNP S5_6240246, which accounted for 26.3% of PVE in C3G and 25.8% in total anthocyanin. Genotypes carrying the minor T allele accumulated twice as much anthocyanin as those carrying the major C allele (**Figure 5C**). In addition, *OsARF3la*, a putative regulatory gene involved in fine-tuning abiotic stress response (Gu et al., 2023), was located 263.67 kb upstream of SNP S5_25298114, which explained 0.8% variation in P3G and 4.3% in total anthocyanin. Lines carrying T alleles exhibited higher anthocyanin content than those with A alleles. Another candidate gene, *OsMYB44*, a potential UV-inducible regulator promoting UV-B tolerance (Zhang et al., 2024), was found 159.27 kb downstream of SNP S9_817645, which showed PVEs of 6.5% for C3G and 6.4% for total anthocyanin. The minor T allele was associated with higher anthocyanin accumulation (**Figure 5E**). Additionally, *OsBZIP71*, a potential UV-inducible anthocyanin regulatory gene involved in abiotic stress tolerance (Liu et al., 2018), was identified 193.22 kb downstream of SNP S9_8066875. Allelic variation at this gene or its nearby regulatory elements could potentially contribute to the 28.5% and 29.3% variation in C3G and total anthocyanin, respectively. The phenotypic means of genotypes with minor A allele were higher than those of genotypes with major T allele (**Figure 5F**).

Notably, most of the 10 lines with highest anthocyanin content under UV conditions carried the favorable alleles of SNPs S5_6240246, S5_25298114 and SNP S9_817645 whereas these alleles were rare among the 10 lines with least anthocyanin content carried one of these alleles (**Supplementary Table S10A**). Except for S5_25298114, this was also mirrored in the whole panel, where the phenotypic means of the genotypes carrying different alleles at these SNPs differed significantly. Similarly, segregation of alleles at SNP S9_8066875 mirrored the phenotypic classes for anthocyanin contents with significantly different mean values. In the subset of 75 genotypes that accumulated more total anthocyanin under UV, the favorable alleles at SNPs S5_6240246, S5_25298114 and S9_817645 had high frequency at 38.9%, 46.7% and 22.7% respectively. In contrast, the favorable allele of SNP S9_8066875 had a frequency of only 6.7% (**Supplementary Table S10B**).

## 4 Discussion

UV exposure has been shown to influence anthocyanin biosynthesis across a wide range of species and tissues (Sharma et al., 2019). Enhanced anthocyanin accumulation in response to UV treatment has been reported in lettuce (Lee et al., 2014), cumin (Ghasemi et al., 2019) and strawberry (Xu et al., 2017). Similarly, anthocyanin biosynthesis in various tissues of rice plants is also activated as a protective mechanism against oxidative stress induced by UV exposure (Hidema and Kumagai, 2006). In black rice, the accumulation of anthocyanin in the grain pericarp not only improve UV tolerance (Zhang et al., 2024) but also provides a great source of antioxidants, underpinning its nutritional and economic value (Das et al., 2023). In this study, a diversity panel of 191 *japonica* black rice genotypes were evaluated under both natural UV exposure and UV-blocked conditions to investigate the genetic regulation of UV-induced anthocyanin accumulation.

The panel was sourced from 11 countries across the Asia-Pacific region, with the largest representation from Indonesia and Philippines (**Supplementary Table S1**). Population structure analysis revealed a degree of substructure within the panel (**Figure 1**), which may reflect underlying geographic differentiation. The clustering of some black rice accessions with white rice accessions may reflect shared ancestry or historical gene flow. There were clear genotype-specific responses to UV exposures within the black rice panel in relation to anthocyanin accumulation. Total anthocyanin levels decreased under UV conditions in 66 genotypes with an average reduction of 43.4 mg/100g seeds while 76 genotypes showed enhanced accumulation with an average increase of 26.4 mg/100g seeds (**Figures 2A–C**). The contrasting patterns indicated that UV response in black rice is not strictly an upregulation of anthocyanin accumulation, but rather a genotype-specific balance between biosynthesis and degradation processes. In some genotypes, UV exposure may trigger higher activity of enzymes involved in anthocyanin degradation, such as poly-phenol oxidase and peroxidase (Hidema and Kumagai, 2006; Zhao et al., 2021), leading to a reduction in final anthocyanin content. The variation also reflected the complexity of anthocyanin metabolism, potentially influenced by grain’s developmental stage and the interplay between anthocyanin biosynthesis and degradation pathways (Thapa et al., 2024). Under both conditions, only weak correlations observed between anthocyanin traits and seed morphology (**Figure 3**) suggested the anthocyanin traits are independent of grain size and shape. This supports the potential to select genotypes with high-anthocyanin content without compromising desirable grain morphology characteristics.

Among four models of GWAS utilized in this study, the two most advanced multi-locus models, BLINK and FarmCPU, demonstrated higher statistical power and better control of false positives (Huang et al., 2019; Wang and Zhang, 2021). These models effectively minimized p-value inflation and highlighted only the most significant SNPs. Several strong candidate genes, including known regulators of anthocyanin biosynthesis and UV response, as well as potential novel genes, were identified in the proximity of the significant SNPs. Notably, SNPs with exceptionally high PVEs include S4_21879467 and S11_19582136 under non-UV conditions, as well as S5_6240246, S5_25298114, S9_8066875 and S11_19582136 under UV conditions (**Table 1**).

Under non-UV conditions, several peak SNP were in linkage with potential candidate genes, mostly structural genes known to be implicated in anthocyanin metabolism. *OsCHS24* was found in the proximity of S11_19582136, which has a high-confidence association with C3G, P3G and total anthocyanin. *OsCHS24* encodes the key chalcone synthase (CHS) isozyme catalyzing the first committed step of the flavonoid pathway. By forming naringenin chalcone from 4-coumaroyl-CoA and 3 malonyl-CoA, *OsCHS24* serves as the central node directing the production of several phenolic compounds like anthocyanins, flavonols and proanthocyanidins (Park et al., 2020). The expression of *CHS* genes has been previously reported to be induced by light or UV exposure in *Arabidopsis* (Jenkins et al., 2001) and rice leaves (Park et al., 2020). The significant association of S11_19582136 with anthocyanin contents under non-UV condition suggests *OsCHS24* contributes to anthocyanin accumulation in rice grains independently of UV induction. Approximately 215kb downstream of S4_21879467, a tetrachlorohydroquinone dehalogenase gene, *OsTCHQD1*, was identified. *OsTCHQD1* encodes glutathione S-transferase (GST) enzyme, which is critical for anthocyanin transportation (Mackon et al., 2024). In ligandin transport mode, GST acts as the main transporter that binds anthocyanins from endoplasmic reticulum to the tonoplast and facilitates the sequestration into the vacuole (Mackon et al., 2021b). The role of *GST* genes in anthocyanin biosynthesis and transport was reported in various crops such as *Bz2* in maize (Marrs et al., 1995), *TT19* in Arabidopsis (Sun et al., 2012) and *GSTU34* in rice (Mackon et al., 2024). With a high PVE of 53.3% to C3G, *OsTCHQD1* may facilitate the vacuolar sequestration of anthocyanin, thereby playing an important role in maintaining anthocyanin stability in rice grains.

Under UV conditions, a different set of peak SNPs were associated with anthocyanin content and proximal candidate genes were found to be involved in the regulation of anthocyanin metabolism. *OsBBX14* was located in the vicinity of SNP S5_6240246, one of the most significant SNPs associated with C3G and total anthocyanin accumulation. *OsBBX14* directly activates *OsC1* or *OsB2*, two key transcription factors regulating the anthocyanin accumulation in rice grains, either independently or in collaboration with *OsHY5* (Kim et al., 2018). It has also been reported to enhance rice photomorphogenesis growth by upregulating *OsHY5L1* under blue light conditions (Bai et al., 2019). In Arabidopsis, *BBX* genes function as either negative or positive regulators of the light-induced anthocyanin biosynthesis (Gangappa and Botto, 2014). Given that SNP S5_6240246 explained a high proportion of phenotypic variance for both C3G and total anthocyanin, and that genotypes carrying the favorable T allele accumulated approximately twice the anthocyanin of those with the C allele (**Figure 5C**), *OsBBX14* stands out as a strong putative candidate gene for UV-regulated anthocyanin production. *OsARF3la* was identified in the proximity of SNP S5_25298114, which was associated with both P3G and total anthocyanin. *OsARF3la* functions within the *miR390*-*TAS3*-*ARF* pathway, a critical regulatory fine-tuning response mechanism to abiotic stress (Gu et al., 2023), suggesting its potential role in regulating anthocyanin biosynthesis as part of protective mechanisms to mitigate UV-induced damage to rice grains.

*OsMYB44* was linked to SNP S9_817645 under UV conditions. Through transcriptomic study, metabolite-based GWAS and gene validation, *OsMYB44* activates the expression of *OsTSβ* and regulate the tryptamine biosynthesis in rice, therefore contributing to the regulation of UV-B tolerance (Zhang et al., 2024). Notably, *MYB44* genes were reported previously to negatively regulate the anthocyanin biosynthesis in a range of crops (Wang et al., 2024). In purple-fleshed sweet potato, *IbMYB44* functions as a repressor of anthocyanin biosynthesis by inhibiting the *IbMYB340*-*IbbHLH2*-*IbNAC56* regulatory complex (Wei et al., 2020), while in non-heading Chinese cabbage, *BcMYB44* negatively controls anthocyanin biosynthesis by suppressing the expression of key structural genes (Hao et al., 2022). Genotypes carrying the major G allele of SNP S9_817645 showed an average anthocyanin reduction of 40% compared to those with minor T allele (**Figure 5E**). The potential role of *OsMYB44* as anthocyanin repressor could be consistent with the differential anthocyanin accumulation in the diversity panel accessions in response to UV treatments, warranting further investigation. A potential UV-inducible anthocyanin regulatory gene *OsbZIP71* was linked to SNP S9_8066875, which shows the highest PVEs for C3G and total anthocyanin under UV conditions. *OsbZIP71* interacts with *OsbZIP73* to reduce ROS accumulations and abscisic acid levels in order to improve abiotic stress tolerance (Liu et al., 2018). The high PVE detected for SNP S9_8066875 emphasized the need for further investigation into *OsbZIP71*, which may play an important role in coordinating responses to multiple abiotic stresses, including UV-B. Notably, the favorable allele of S9_8066875 was rare with a frequency of only 8.1% (**Supplementary Table S9**). Genotypes with favorable A allele exhibited a strong increase in anthocyanin accumulation compared to those with G allele, with an average increase of 93% (**Figure 5F**), making it a promising target for marker development and varietal improvement in breeding programs focused on enhanced UV resilience and anthocyanin content.

Although UV-dependent anthocyanin phenotypes were variable within the investigated diversity set (**Figure 2**), the lack of overlapping in SNP peaks between non-UV and UV conditions suggested that UV exposure affects the regulation of anthocyanin pathways. Interestingly, significant SNPs detected under non-UV conditions were linked to structural genes of anthocyanin biosynthesis (*OsCHS24, OsTCHQD1)*, while regulatory genes (*OsBBX14*, *OsARF3la*, *OsMYB44* and *OsbZIP71*) were found near SNPs under UV conditions. This suggests that in the absence of UV, baseline anthocyanin accumulation is primarily determined by variation in structural genes of biosynthesis pathway. Contrastingly, under UV exposure, the observed differences in anthocyanin accumulation seems to be driven by sequence differences in regulatory components of these pathways. Three peak SNPs detected under UV conditions (**Figures 5C, E, F**) showed significant association with anthocyanin content, indicating the effect of UV on the anthocyanin regulation. Of the three regulatory candidates linked with these peaks, two gene, *OsBBX14* and *OsMYB44* have been reported previously to be induced by light or UV (Bai et al., 2019; Zhang et al., 2024) and to participate in anthocyanin regulation (Kim et al., 2018; Wang et al., 2024). Favorable alleles at these loci occurred at high frequencies within the subset of genotypes showing enhanced anthocyanin accumulation under UV (**Supplementary Table S10B**). Therefore, these alleles appear to contribute to an increased capacity for anthocyanin accumulation under UV exposure.

Despite the growing research interest in pigmented rice, only a handful of GWAS studies have been conducted to reveal the genetic basis of rice grain pigmentation. These studies primarily investigated pericarp color parameters (Kiran et al., 2025; Mbanjo et al., 2023; Wang et al., 2023) or total flavonoid content as an antioxidant trait (Purnama et al., 2025). However, limitation in these studies stem from either the use of less powerful GWAS models, such as MLM (Purnama et al., 2025; Wang et al., 2023), or the use of phenotyping methods that rely on proximate or predicted data (Kiran et al., 2025). Among the SNPs detected in these studies, the most significant ones were situated near the *Rc* gene, a key regulator of proanthocyanidin biosynthesis and red pigmentation in the pericarp (Sweeney et al., 2006). The association study by Mbanjo et al. (2023) included 48 genotypes that overlapped with the diversity panel in the present study; however, it did not identify similar candidate genes. Of the two SNPs associated with anthocyanin content estimated using multi-spectral phenotyping, S01_28051821 was linked with Os01g0681000, a gene encoding wax synthase (Mbanjo et al., 2023), though its connection to anthocyanin biosynthesis remains unclear. The current study is the first GWAS study to employ HPLC for precise quantification of anthocyanins in grain pericarps, thus providing more accurate phenotype data for association analysis. This phenotyping approach, combined with the use of two of the most advanced GWAS models, enabled the identification of several novel structural and regulatory genes in the anthocyanin biosynthesis associated with variations in rice grain pigmentation. Moreover, it is the first study to propose specific UV-dependent regulatory mechanism of rice grain pigmentation.

An important limitation of the present study is that microclimatic parameters other than UV-B radiation were not measured under the UV-blocking structure. The polycarbonate roofing could have introduced unintended environmental effects, such as reduced airflow or altered temperature slightly, particularly under low-wind conditions, which could have unknown influence on the anthocyanin biosynthesis pathway. Therefore, the observed differences between the UV-blocked and ambient-UV conditions should not be attributed exclusively to UV-B radiation. In addition, the experiment was conducted at a single location and in a single season, and multi-season and multi-location experiments would be required to determine the stability and reproducibility of these responses and genetic associations. Future experiments could minimize these potential confounding effects by using controlled glasshouse conditions with regulated temperature and airflow. Distinct UV treatments, including reduced, ambient, and elevated UV exposure, could also be established using controlled lighting configurations. Such experiments would help disentangle the specific contribution of UV-B from other environmental factors. In addition, the functions of the candidate genes identified through GWAS require further validation to increase confidence in their potential roles in UV-responsive anthocyanin accumulation. Such validation was beyond the scope of the present study and could include targeted gene expression analysis, such as quantitative PCR, and functional genetic approaches, including gene knockout using CRISPR-Cas9.

## 5 Conclusion

Our study is the first to explore the genetic basis of grain pigmentation in response to natural UV-B exposure. By identifying both known and proposing regulatory genes involved in anthocyanin biosynthesis, grain color and UV-B response, this study provides a valuable foundation for future functional validations and variety improvement. In particular, *OsBBX14*, *OsMYB44* and *OsbZIP71* emerge as promising candidates for marker development, with potential applications in breeding programs aimed at enhanced nutritional content and improved UV-B tolerance. These findings not only advance the understanding of the molecular regulatory mechanisms governing grain pigmentation under UV exposure, but also create new opportunities for the development of climate-resilient, nutrient-rich rice varieties.

## Data Availability

The datasets presented in this study can be found in online repositories. The names of the repository/repositories and accession number(s) can be found in the article/**Supplementary Material**.
